# Single-cell transcriptomics identifies an increased Ly6G- neutrophil population and accentuated T-cell cytotoxicity in tobacco flavored e-cigarette exposed mouse lungs

**DOI:** 10.1101/2025.02.17.638715

**Published:** 2025-08-07

**Authors:** G Kaur, T Lamb, A Tjitropranoto, Irfan Rahman

**Affiliations:** 1 Department of Environmental Medicine, University of Rochester Medical Center, Rochester, NY, USA

**Keywords:** E-cigarettes, Tobacco, scRNA seq, Immune cells, Innate immunity, Neutrophils, T-cells

## Abstract

E-cigarettes (e-cigs) are a public health concern for young adults due to their rising popularity despite evidence of harmful effects, i.e. increased oxidative stress and immunotoxicity. Yet an extensive study defining the cell-specific immune changes upon exposure to flavored e-cigs remains elusive. To determine the immunological lung landscape upon acute nose-only exposure of C57BL/6J to flavored e-cig aerosols, we performed single-cell RNA sequencing (scRNA seq). scRNA profiles of 71,725 cells were generated from control and treatment groups (n=2/sex/group). An increase in the Ly6G- neutrophil population was identified in lungs exposed to tobacco flavored e-cig aerosol. Differential gene expression analyses identified dysregulation of T-cell mediated pro-inflammation (*Cct7*, *Cct8*) and stress-response signals (*Neurl3*, *Stap1*, *Cirbp* and *Htr2c)* in the lungs of mice exposed to e-cig aerosols, with pronounced effects for tobacco flavor. Flow cytometry analyses and cytokine/chemokine assessments within the lungs corroborate the scRNA seq data, demonstrating an increase in the T-cell percentages and levels of T-cell associated cytokine/chemokines in the lungs of tobacco-flavored aerosol exposed mice. Increased levels of *Klra4* and *Klra8* expression also suggest an enhanced natural killer (NK) cell activity in this mouse group. Interestingly, the increase in the level of Ly6G – (immature) neutrophils upon exposure to tobacco-flavored e-cig aerosols was noticed for female C57BlL6J mice and not their male counterparts which was validated through immunofluorescence staining. Overall, this study identifies sex-specific increase in the percentages of Ly6G- neutrophils that may be responsible for dampened innate immune responses in females and heightened T-cell cytotoxicity in male mouse lungs of tobacco-flavored e-cig aerosol exposed mice.

## Introduction

Electronic cigarettes (e-cigs) or electronic nicotine delivery systems are a relatively novel set of tobacco/nicotine and flavored products that have gained immense popularity amongst adolescents and young adults in many countries including the United States (US), the United Kingdom (UK) and China. Flavors are one of the key features that make these products alluring to the younger diaspora (1). Reports indicate that in 2020 about 22.5% of high school students and 9.4% of middle school students in the US were daily vapers or e-cig users with fruit (66%), mint (57.5%), and menthol (44.5%) being the most commonly used flavors (2). However, not much is known about the flavor-specific effects of e-cig vaping on the health and immunity of an individual specially focusing on cell-types and gene transcripts.

E-cig products and aerosols are known to contain harmful constituents including formaldehyde, benzaldehyde, acrolein, n-nitrosamines, volatile organic compounds (VOCs), ketenes, and metal ions (3–5). Studies have indicated that exposure to e-cigs may enhance inflammatory responses, oxidative stress, and genomic instability in exposed cells or animal systems (6–8). Risk assessment (systemic) of inhaled diacetyl, a potential component of e-liquids, has estimated the non-carcinogenic hazard quotient to be greater than 1 amongst teens (9). Furthermore, clinical and *in vivo* studies have suggested that exposure to e-cig aerosols could impair innate immune responses in the host thus making them more susceptible to bacterial/viral infections. The bacterial clearance, mucous production, and phagocytic responses in these individuals are shown to be affected upon use of e-cigs (10–14).

However, cell-specific changes within the lung upon vaping is not fully understood, making it hard to determine the long-term health impacts of the use of these novel products. In this respect, single cell technology is a powerful tool to analyze gene expression changes within cell populations to study cell heterogeneity and function (15–17). Such an investigation is important to deduce the health effects of acute and chronic use if e-cigs in young adults. In this study, we aim to determine effects of e-cig exposure on mouse lungs at single cell level. To do so, we exposed C57BL/6J mice to 5-day nose-only exposure to air, propylene glycol: vegetable glycerin (PG:VG), fruit-, menthol- and tobacco-flavored e-cig aerosols. The nose-only exposure has more translational relevance over whole-body exposure (18), owing to which, we chose nose-only exposure profile for this work. To limit the stress to the animals a 1-hour exposure was chosen per day. We performed single-cell RNA sequencing (scRNA seq) on the lung digests from exposed and control animals and identified neutrophils and macrophages, among others, as the major cell populations in the lung that were affected upon acute exposure. We were able to identify 29 gene targets that were commonly dysregulated amongst all our treatment groups upon aggregating results from the major lung cell types identified in this study. These gene targets are the markers of early immune dysfunction upon e-cig aerosol exposure *in vivo* and could be studied in detail to understand temporal changes in their expression that may govern allergic responses and adverse pulmonary health outcomes upon acute and sub-acute exposures to e-cigarette aerosols.

## Results

### Exposure to e-cig aerosol results in flavor-dependent exposure to different metals and mild histological changes in vivo.

**Figure 1:**
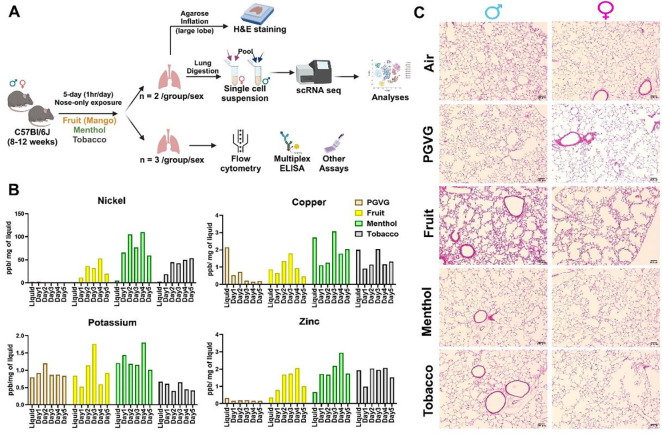
Flavor-dependent changes in the levels of quantified metals, but no major histological damage on acute exposure to flavored e-cig aerosol in mice. Schematics showing the exposure profile and experimental design to understand the effect of exposure to differently flavored e-cig aerosols in the lungs from C57BL/6J mice using scRNA seq **(A)**). Bar Graph showing the levels of metals in the aerosols captured each day of exposure using ICP-MS **(B)**. Lung morphometric changes as observed using H&E staining of lung slices from air, PG:VG and differently flavored e-cig exposed mice. Representative images of n = 2/sex/group at 10X magnification is provided **(C)**.

This study was designed to characterize the effects of exposure to flavored e-cig aerosols at single-cell level to understand the immunological changes in the lung microenvironment upon e-cig use. To do so, we generated a first ever single-cell profile of e-cig aerosol exposed mouse lungs (n= 2/sex/group). The thus obtained results were then validated with the help of our validation cohort of n= 3/sex/group as shown in Fig 1A. Since all the commercially available e-liquids used in this study contained tobacco derived nicotine, we first determined the level of serum cotinine (a metabolite of nicotine) to prove successful exposure to the mice in each treatment group. As expected, we did not see any traces of cotinine in the serum of air and PG:VG exposed mice. Significant levels of cotinine were detected in the serum of mice exposed to fruit-, menthol-, and tobacco-flavored e-cig aerosols **(Figure S1B)**.

Since metals released upon heating of the coils of e-cig devices are a source of toxicity upon vaping (19, 20), we further monitored the levels of metals in the e-cig aerosols generated during each day of mouse exposures. This acted as an indirect measure of characterizing the chemical properties of the aerosols used for exposure in this study. To monitor the release of metals into the lungs of the animals, the aerosol condensate from each day of exposure was collected and the levels of select elements were detected using ICP-MS. A detailed account of the concentrations of identified elements/metals is provided in Table 1. Interestingly, we identified flavor-dependent changes in the levels of metals like Ni, Zn, Na, K and Cu. Note, despite the use of same wattage and temperature (max of 230°C) for generation of e-cig aerosols, the leaching of each metal varied per day of exposure (Figure 1B). This is a crucial result as it highlights the importance of studying the impacts of coil composition and device design on the chemical composition of generated e-cig aerosols. These variations might affect the risk and toxicity associated with each of these products, an area that warrants further study.

Next, we performed H&E staining of the lung tissue sections to study the morphometric changes in the mouse lungs upon exposure to differently flavored e-cig aerosols. We did not find much evidence of tissue damage or airspace enlargement upon acute exposures in our model, as expected. However, we found evidence of increased alveolar septa thickening in the lungs of both male and female mice exposed to fruit-flavored e-cig aerosols (Figure 1C). However, this could be a result of lower agarose inflation observed for the mouse lungs in this group. We had challenges/difficulties with inflating the mouse lungs for all mice in this group, however male mice showed more restriction than the female. Owing to the lack of proper inflation, the lungs were in a collapsed state than the other groups which could be a probable cause of the histological observations. Airway obstruction could be indicative of “atelectasis”, a condition that may result from lack of surfactant or alveolar damage thus affecting the lung expansion in these mice. Since this was not the prime focus of this study, we did not conduct further experiments to confirm our speculations, but nevertheless, we report these changes to inform and encourage further research in this area.

### Detailed map of cellular composition during acute exposure to e-cig aerosols reveals distinct changes in immune cell phenotypes.

As the principle focus of this study was to identify the flavor-dependent and independent toxicities upon exposure to commercially available e-cig aerosols at the single-cell level, we performed the scRNA seq on the mouse lungs from exposed and control mice. After quality control filtering **(Figure S2),** normalization and scaling, we generated scRNA seq profiles of 71,725 cells in total. Except for the PG:VG group, all the rest of the treatments had approximately similar cell viabilities, cell capture, and other quality assessments in this study. However, for uniform analyses, equal features/ genes were used across all the groups for subsequent analyses. A detailed account of the cell viability, number of samples (single-cell capture), and gene features identified before and after filtering upon QC check of sequenced data is provided in **Supplementary File S1**.

**Figure 2:**
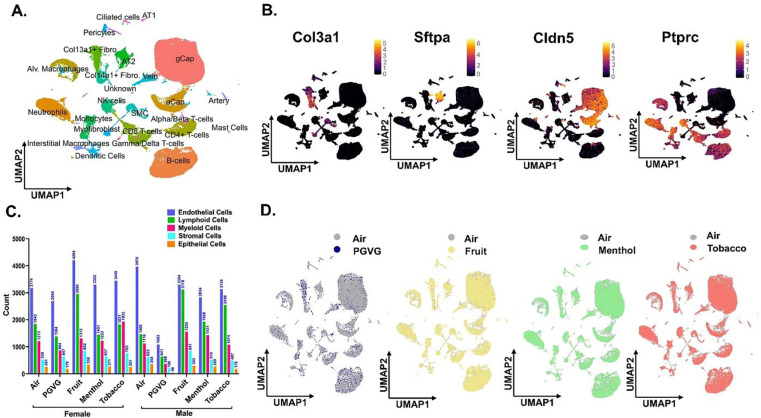
scRNA seq analyses reveal maximum changes in the cell states of immune cell population upon exposure to differently flavored e-cig aerosols. Male and female C57BL/6J mice were exposed to 5-day nose-only exposure to differently flavored e-cig aerosols. The mice were sacrificed after the final exposure and lungs from air (control) and differently flavored e-cig aerosol (fruit, menthol, and tobacco)-exposed mice were used to perform scRNA seq. UMAP plot of 71,725 cells captured during scRNA seq showing the four major cell populations identified from control and experimental mouse lungs **(A)** and the expression of canonical markers used for identifying stromal (*Col3a1*), epithelial (*Sftpa1*), endothelial (*Cldn5*) and immune (*Ptprc*) cell populations. The intensity of expression is indicated by the red-yellow coloring **(B)**. The cell frequencies (plotted as counts) of different cell clusters in each sample type showing the sex-dependent variations in the cell composition on exposure to differently flavored e-cig aerosols **(C)**. Group-wise comparison of the UMAPs upon comparing PG:VG (blue), fruit (yellow), menthol (green) and tobacco (red) versus air (air) groups following dimensionality reduction and clustering of scRNA seq data **(D)**. Here, AT1: alveolar type I, AT2: alveolar type II, SMC: smooth muscle cell, gCap: general capillary, aCap: alveolar capillary, and NK: natural killer.

Uniform Manifold Approximation and Projection (UMAP) was used for dimensionality reduction and visualization of cell clusters. Cell annotations were performed based on the established cell markers in Tabula Muris database and available published literature and we identified 24 distinct cell clusters as shown in Figure 2A. The general clustering of individual cell types based upon the commonly known cell markers was used to identify -**Endothelial** (identified by expression of *Cldn5*), **Epithelial** (identified by expression of *Sftpa1*), **Stromal** (identified by expression of *Col3a1*), and **Immune** (identified by expression of *Ptprc*) cells (Figure 2B). The ‘FindVariableFeatures’ from Seurat was used to identify cell-to-cell variation between the identified clusters and the top 2000 variable genes identified in each cluster have been elaborated in **Supplementary File S2**. Upon plotting the number of counts/cells identified in each cluster in a sex- and treatment-specific manner we observed minor changes, though not significant, in the count of myeloid and lymphoid cell clusters in e-cig aerosol exposed mouse lungs as compared to air controls (Figure 2C). A detailed account of the two-way ANOVA statistics for individual cell types is provided in **Supplementary File S3A**.

Groupwise comparisons of each treatment group versus Air control, did not show many changes in the cell clusters thus showcasing little to no effect on the overall lung cellular compositions in treated and control groups. However, the number of cells identified in each cluster for PG:VG treatment group was very low. We would like to report that this is an outcome of the low viability of this sample prior to scRNA seq, and not necessarily the effect of the treatment (Figure 2D). Differential gene expression analyses showed dysregulation of genes in all cell populations, but maximum effect was observed on the cells from immune cell clusters. This is not surprising, as the immune system, especially the myeloid cells form the frontline of the host’s defenses against external stressors (21, 22). Compared to air, we observed a dysregulation of 553 genes (338 upregulated; 215 downregulated) in the myeloid cell cluster of mouse lungs exposed to tobacco-flavored e-cig aerosol. We identified 324 and 24 DEGs in the myeloid lung cluster from mouse lungs exposed to menthol- and fruit-flavored e-cig aerosols respectively as compared to air control **(Supplementary Files S5-S7)**. For the lymphoid cluster, we observed maximum dysregulation in the lungs exposed to fruit-flavored e-cig aerosols with a total of 112 DEGs. In contrast, 41 and 9 significant DEGs were identified for lymphoid cluster from lungs exposed to tobacco and menthol-flavored aerosols respectively **(Supplementary Files S9-S11)**.

It is important to mention here that most of the commercially available e-liquids /e-cig products use PG:VG as the base liquid to generate the aerosol and act as a carrier for flavoring chemicals. Thus, we compared the effect of PG:VG alone in our study to make further comparisons between individual flavoring products with that of PG:VG only. DESeq2 analyses showed dysregulation of 276 genes in mouse lungs exposed to PG:VG alone in the myeloid cell cluster as compared to air controls **(Supplementary Files S4)**. Contrary to this, exposure to PG:VG aerosols affected 24 genes in the lymphoid cluster as compared to air control **(Supplementary Files S8)**. Furthermore, when compared to PG:VG, exposure to fruit, menthol and tobacco-flavored e-cig aerosol dysregulated 262, 873 and 960 genes in the myeloid cluster and 37, 64 and 112 genes in the lymphoid cluster respectively. A detailed account of the DEGs identified upon comparing fruit, menthol and tobacco-flavored e-cig aerosol exposed mouse lungs to PG:VG in myeloid and lymphoid clusters have been provided in **Supplementary Files S13-S18.**

### Exposure to differently flavored e-cig aerosols results in sex-specific variations in neutrophilic and eosinophilic response in the mouse lungs.

**Figure 3:**
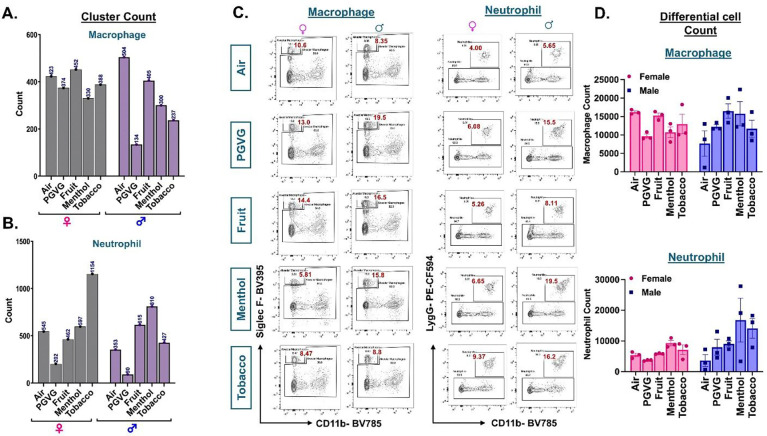
Cellular composition of myeloid cells in air and e-cig aerosol exposed mouse lung reveal sex-specific increase in neutrophil count through scRNA seq but not flow cytometry. Cell frequencies (represented as counts) of alveolar macrophages **(A)** and neutrophils **(B**) across controls and flavored e-cig aerosol exposed mouse lungs were plotted in a sex-specific manner. Representative flow plots **(C)** and bar graphs **(D)** showing sex-dependent changes in the percentages of neutrophils (CD45+ CD11b+ Ly6G+) and alveolar macrophages (CD45+ CD11b− SiglecF+) populations in lung digests from mice exposed to differently flavored e-cig aerosols. Data are shown as mean ± SEM (n = 3/sex/group).

We found dysregulation in immune cell population, more specifically the myeloid cells, upon exposure to e-cig aerosols through our previous analyses. We thus plotted the changes in cell counts across the various treatment groups and control in a sex-dependent manner. scRNA seq results did not show any change in the cell frequencies of alveolar macrophages in flavored e-cig aerosol exposed mouse lungs as compared to controls (Figure 3A; **Supplementary File S3B**). However, the cell count of neutrophils in tobacco-flavored e-cig aerosol exposed mice lungs were found to be increased in females, but not in males (Figure 3B, **Supplementary File S3B**). Taken together, we show a significant shift towards the neutrophil-mediated immune response in the lungs of female mice exposed to tobacco-flavored e-cig aerosols. We used flow cytometry to validate the scRNA seq changes. While the changes observed in the levels of alveolar macrophages in the mouse lungs corroborated with our scRNA seq findings, the results for the neutrophil cell cluster did not show matching trend. This could be due to the increase in the levels of immature neutrophils in the lungs of female tobacco-e-cig exposed mouse lungs characterized by the absence of Ly6G cell surface marker, which was used to stain for neutrophils via flow cytometry (Figure 3C–D, **Supplementary File S3D**).

It is important to mention that due to the presence of nicotine in all the e-liquids used as treatment groups for this study, we added an extra control group- PG:VG+Nic− for select experiments. Flow cytometric analyses revealed slight variations in the lung neutrophils and macrophage percentages observed in PG:VG+Nic group as compared to PG:VG only. However, none of these changes were significant. Furthermore, the patterns of change observed for the lungs exposed to aerosols from flavored e-liquids were quite distinct from those observed for PG:VG+Nic thus proving that the observed changes are not solely due to the presence of nicotine in these groups **(Figures S3A-B)**.

**Figure 4:**
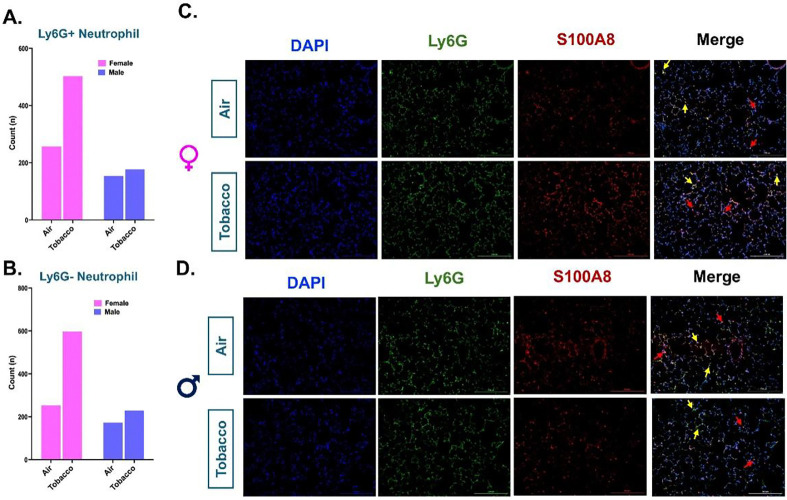
Co-immunofluorescence validates the increase of Ly6G− and Ly6G+ neutrophil population in the tobacco flavored e-cig exposed female C57BL/6J mice. The myeloid cell clusters were subsetted to identify two populations of neutrophils with and without the presence of Ly6G cell marker representing mature and immature neutrophils respectively **(Figure S3C)**. Bar graph showing the sex-specific changes in the cell frequencies (denoted as counts) of Ly6G+ **(A)** and Ly6G− **(B)** neutrophils in the lung of tobacco-flavored e-cig aerosol exposed mouse lung as compared to air control. scRNA seq findings were validated by staining the tissue sections from tobacco-flavored e-cig aerosols and control (air) with Ly6G (green) and S100A8 (red, pan-neutrophil marker). Representative images showing the co-immunostaining of Ly6G and S100A8 (shown as yellow puncta) in female **(C)** and male **(D)** control and tobacco-flavored e-cig aerosol exposed mice. Here Ly6G+ neutrophils are represented by double staining using yellow arrows, while Ly6G− neutrophils are denoted by red arrows.

To probe further, we subclustered the myeloid cell populations. Upon sub clustering, we identified 14 unique clusters comprising all the major cell phenotypes including neutrophils, alveolar macrophages (AM), interstitial macrophages (IM), monocytes, dendritic cells (DC), and mast cells (**Figure S3C)**. A detailed account of all the cell types identified with their respective marker genes is provided in **Supplementary File 12**. On deeper evaluation, we identified two unique phenotypes of neutrophils (identified by *S100a8*, *Cxcl2*, *Sell* and *Lcn2)* in the mouse lungs. These clusters were named as Ly6G^+^ Neutrophils and Ly6G^−^ Neutrophils based on the presence or absence of Ly6G marker, respectively **(Figure S3C)**. Ly6G is important for neutrophil migration, maturation and function within the lung (23). Interestingly we found an increase in the levels of both Ly6G+ and Ly6G− neutrophil in the tobacco-flavored e-cig aerosol exposed female mouse lungs as compared to air control. Though we did not observe similar changes in male mice exposed to tobacco-flavored e-cig aerosol (Figure 4A–B, **Supplementary File S3C**). The scRNA seq findings were further validated using co-immunofluorescence using S100A8 (red, pan-neutrophil marker) and Ly6G (green, marker of mature neutrophil). Co-immunofluorescence results show increase in levels of both Ly6G + (yellow stained) and Ly6G – (red stained) neutrophil in female mice exposed to tobacco flavored e-cig aerosol, but not males further corroborating our previous findings and suggesting that sex-specific changes in innate immune response in vivo (Figures 4C–D).

Though we did not find a distinct eosinophil cluster through our scRNA seq analyses, a significant decline in the level of eosinophils in the lungs of female mice exposed to menthol-flavored e-cig aerosol was observed through flow cytometric analyses. Though not significant, marked decline in the eosinophil count was observed upon acute exposure of male and female C57BL/6J mice to tobacco-flavored e-cig aerosols. Contrarily, the eosinophil count in the lung of male mice exposed to fruit flavored e-cig aerosols was observed to be significantly higher as compared to PG:VG exposed lungs **(Figures S4)**.

### Activation of T-cell cytotoxic responses in lymphoid cells upon exposure to tobacco-flavored e-cig aerosols.

**Figure 5:**
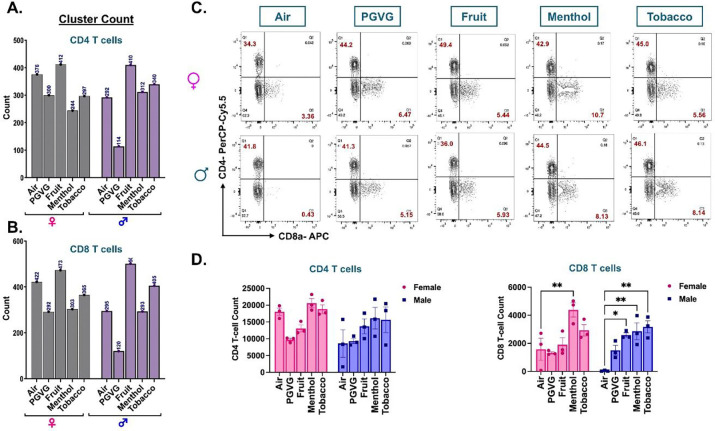
scRNA seq analyses and flow cytometry result show flavor-dependent increase in CD8 T cells in lungs of differently flavored e-cig aerosol exposed C57BL/6J mouse. The cell frequencies (denoted as counts) of CD4 **(A)** and CD8 **(B)** T-cells showing the sex-dependent variations in the cellular composition upon exposure to differently flavored e-cig aerosols. Representative flow plots **(C)** and bar graph **(D)** showing changes in the mean counts of CD4+ and CD8+ T-cells in the lung tissue digest from male and female mice exposed to differently flavored e-cig aerosols as determined using flow cytometry. Data are shown as mean ± SEM (n = 3/sex/group). *p<0.05, and **p<0.01, per Tukey post hoc two-way ANOVA comparison.

We next studied the changes in the lymphoid clusters of treated and control samples. Both scRNA seq and flow cytometric analyses showed no variation in the CD4+ T cell frequency in the lungs of different e-cig flavored exposed mouse lungs as compared to air controls. We also identified flavor-dependent variations in the cell frequencies of CD8 T cells (p= 0.0802) in the lymphoid cluster from mouse lungs (Figures 5A–B, **Supplementary File S3B**). Flow cytometric analyses corroborated with the scRNA seq findings and showed a significant increase in the CD8+ T cell percentages in the lungs of tobacco-flavored e-cig aerosol exposed mice lungs as compared to air control in male mice (Figure 5C–D, **Supplementary File S3D**). Of note, we found a significant increase in the CD4+ T cell percentages in menthol flavored e-cig aerosol exposed mouse lung as compared to PG:VG exposed control **(Figure S5)**.

### Exposure to fruit-flavored e-cig aerosol affected the mitotic pathway genes in the lymphoid cluster

**Figure 6:**
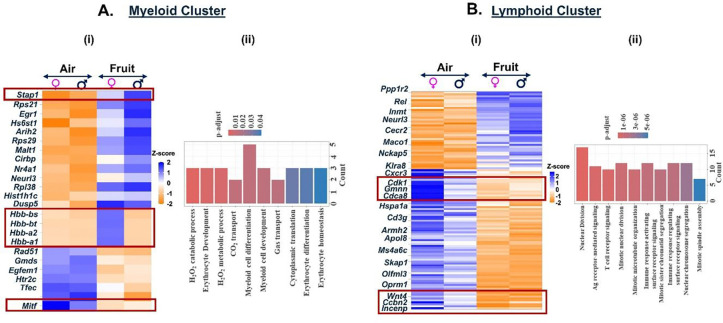
Exposure to fruit flavored e-cig aerosols result in activation of oxidative stress-mediated innate immunity in C57BL/6J mouse lungs. Male and female C57BL/6J mice were exposed to 5-day nose-only exposure to fruit-flavored e-cig aerosols. The mice were sacrificed after the final exposure and mouse lungs from air (control) and aerosol (fruit-flavored) exposed groups was used to perform scRNA seq. Heatmap and bar plot showing the DESeq2 **(i)** and GO analyses **(ii)** results from the significant (p<0.05) DEGs in the myeloid **(A)** and lymphoid **(B)** cell cluster from fruit-flavored e-cig aerosol exposed mouse lungs as compared to controls.

To assess the effects of acute exposures to differently flavored e-cig aerosols on the cell composition in the mouse lungs, we performed differential expression analyses for each flavor in comparison to air and PG:VG controls. The mango-flavored e-cig aerosol exposure had the mildest effect on the cell compositions and gene expression as compared to the controls in our study. We did not observe major dysregulation in the gene expression for myeloid cell cluster in fruit-flavored e-cig exposed mouse lungs as compared to air controls. A total of 24 genes (17 upregulated; 7 downregulated) were differentially expressed in the myeloid clusters in lungs of animals exposed to fruit-flavored e-cig aerosols (Figure 6Ai, **Supplementary File S5A**). GO analyses identified terms like ‘myeloid cell differentiation’, gas transport’ and ‘H2O2 catabolic process’ as the top hits for this cluster (Figures 6Aii, **Supplementary File 5B**). Importantly, few gene clusters (*Hbb-bs*, *Hbb-bt*, *Hba-a2* and *Hbb-a1*) showed sex-specific changes in the gene expressions in exposed lungs as compared to control. These gene clusters enrich for ‘erythrocyte development’ upon GO analyses and warrant further study.

We further found dysregulation of 112 genes (40 up- and 72-down-regulated) in mouse lungs exposed to fruit-flavored e-cig aerosols in the lymphoid cluster. Dysregulation of genes (*Incenp*, *Wnt4*, *Ccnb2*, *Trat1*, *Themis*) enriched for ‘T-cell receptor signaling’ and ‘nuclear division’ were observed in exposed group as compared to air control (Figure 6Bi, **Supplementary file 9A**). Upregulation of genes like *Malt1* (Mucosa-associated lymphoid tissue lymphoma translocation protein 1), *Ppp1r2* (Protein phosphatase inhibitor 2), and *Spib* (Transcription factor Spi-B) indicates activation of T-cell mediated immune response upon exposure to fruit-flavored aerosol (24–26). GO analyses showed terms like ‘mitotic nuclear division’, ‘mitotic spindle assembly’, and ‘nuclear chromosome segregation’ to be highly affected on exposure to fruit-flavored e-cig aerosol in C57BL/6J mouse lungs (Figure 6Bii, **Supplementary File 9B**).

### Exposure to Menthol-flavored e-cig aerosols affected the immune cell function

**Figure 7:**
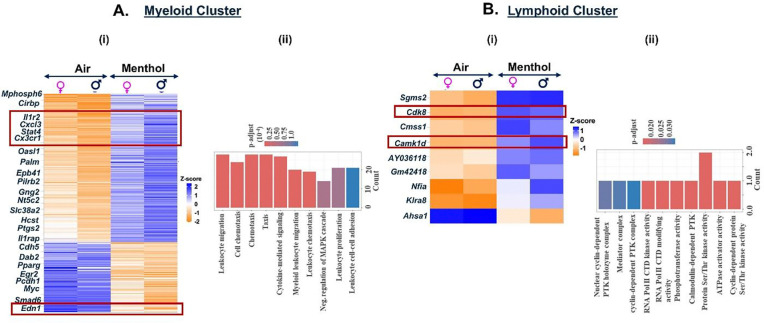
Exposure to Menthol flavored e-cig aerosols result in activation of innate immune responses C57BL/6J mouse lungs. Male and female C57BL/6J mice were exposed to 5-day nose-only exposure to menthol-flavored e-cig aerosols. The mice were sacrificed after the final exposure and mouse lungs from air (control) and aerosol (menthol-flavored) exposed groups was used to perform scRNA seq. Heatmap and bar plot showing the DESeq2 **(i)** and GO analyses **(ii)** results from the significant (p<0.05) DEGs in the myeloid **(A)** and lymphoid **(B)** cell cluster from menthol-flavored e-cig aerosol exposed mouse lungs as compared to controls.

We demonstrated an upregulation of 220 genes and a downregulation of 104 genes in the myeloid cluster of mouse lungs exposed to menthol-flavored e-cig aerosol as compared to ambient air. We observed increased expression of inflammatory genes including *Cxcl3*, *Il1r2, Stat4*, pointing towards the activation of chemokine-mediated signaling due to exposure to menthol-flavored e-cig aerosol. We also found a significant decrease in the expression of *Edn1* (endothelin1) in the myeloid cells of flavored e-cig aerosols as compared to air controls. These genes play a role in positive regulation of neutrophil recruitment and inflammatory responses into the lungs (27–31). GO analyses of the DEGs (p<0.01), thus, showed ‘cytokine-mediated signaling’, ‘cell chemotaxes’, and ‘negative regulation of MAPK cascade’ as the top hits as shown in Figure 7A (**Supplementary File 6A-B**).

Contrary to the responses observed for exposure to fruit-flavored e-cig aerosols, we found significant upregulation in the expression of *Cdk8* and *Camk1d* genes in the lymphoid cell populations for menthol-flavored aerosol exposed mouse lungs (Figure 7B, **Supplementary File 10A**). *Cdk8* (cyclin-dependent kinase 8) is a transcriptional regulator that has a role in the cell cycle progression (32). Whereas *Camk1d* (calcium/calmodulin dependent protein kinase ID) functions to regulate calcium-mediated granulocyte function and respiratory burst within the cells(33). Taken together, our results point towards an increase in cell proliferation and gene transcription in the lymphoid cluster of mouse lungs exposed to menthol-flavored e-cig aerosols as indicated by enrichment of terms like ‘cyclin-dependent protein serine/threonine kinase activity’, RNA polymerase II CTD modifying activity’ and ‘calmodulin-dependent protein kinase activity’ associated with this cell cluster (Figure 7Bii, **Supplementary File 10B**).

### Exposure to Tobacco-flavored e-cig aerosol elicits immune response in myeloid cell and cell cycle arrest in lymphoid cell population

**Figure 8:**
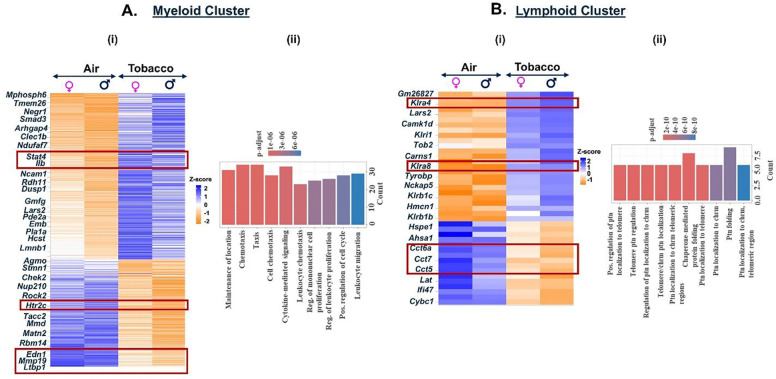
Exposure to Tobacco flavored e-cig aerosols result in activation of cytolysis and neutrophil chemotaxis in C57BL/6J mouse lungs. Male and female C57BL/6J mice were exposed to 5-day nose-only exposure to tobacco-flavored e-cig aerosols. The mice were sacrificed after the final exposure and mouse lungs from air (control) and aerosol (tobacco-flavored) exposed groups was used to perform scRNA seq. Heatmap and bar plot showing the DESeq2 **(i)** and GO analyses **(ii)** results from the significant (p<0.05) DEGs in the myeloid **(A)** and lymphoid **(B)** cell cluster from tobacco-flavored e-cig aerosol exposed mouse lungs as compared to controls.

Like menthol-, tobacco-flavored e-cig aerosol also elicited a significant increase in the expression of 338 genes and a decrease in 215 genes as compared to air controls in the myeloid cell cluster. We observed an increase in the expression of chemokines like *Stat4*, *Il1b*, and *Il1bos* in the myeloid cells resulting in terms like ‘cytokine-mediated signaling pathway’ enriched upon functional annotation of DEGs. Like menthol-exposed mouse lungs, we found significant downregulation of Edn1 gene in the myeloid cluster in lungs of mice exposed to tobacco-flavored e-cig aerosol (Figure 8A, **Supplementary File 7A-B**).

We also observed a downregulation of genes responsible for chaperone-mediated protein folding (*Cct5*, *Cct7*, *Cct8*) in the lymphoid cells from tobacco-flavored e-cig aerosol exposed mouse lungs. Downregulation of these genes could be indicative of the accumulation of misfolded proteins in these lungs which may lead to enhanced cell death (34, 35). In fact, we found increased expression of killer cell lectin-like receptor (*Klra*)-4 and 8 in exposed mice lungs as compared to air controls, thus indicating that upon exposure to tobacco-flavored e-cig aerosols, the lymphoid cells undergo protein misfolding thereby resulting in increased cell death (Figure 8B, **Supplementary File 10A-B**).

### Dysregulation of chemokine signaling and T-cell activation on exposure to flavored e-cig aerosols.

Since we showed increased production of cytokines/chemokines, driving the immune responses in mouse lungs exposed to flavored e-cigs, we performed multianalyte assay to determine the levels of these inflammatory cytokines in the lung digests from the exposed animals as shown in **Figure S3D**. Exposure to tobacco-flavored e-cig aerosol resulted in a marked increase in the levels of chemotactic chemokines including CXCL16, CXCL12, CXC3R and proinflammatory cytokines including CCl12, CCL17, CCL24, and Eotaxin in the mouse lung digests as compared to air control. Supporting our transcriptional data, the levels of IL1b were dampened in the exposed mouse lungs thus showing negative regulation of IL-1 mediated signaling. We identified sex-dependent changes in the cytokine levels in lung digests from fruit-flavored e-cig aerosols with male mice showing more dampening of cytokine/chemokine protein levels as compared to their female counterparts which aligns with lowed innate immune responses driven by neutrophils and macrophages in the males. Interestingly, the fold changes in the PG:VG+Nic group were contrasting to those observed by PG:VG alone and flavored e-cig aerosol exposed mouse lungs, again supporting our previous deduction that the observed changes are observed as a concerted effect of chemical present in the e-liquid used for flavored e-cig aerosol exposure.

To identify genes that were commonly dysregulated upon exposure, we generated a list of common genes that were significantly dysregulated in exposure categories (fruit, menthol and tobacco). We identified 9 such target genes-Ne*url3*, *Egfem1*, *Stap1*, *Tfec*, *Mitf*, *Cirbp*, *Hist1h1c*, *Gmds,* and *Htr2c* that were dysregulated in the myeloid cluster from lungs exposed to differently flavored e-cig aerosol, but not PG:VG. We observed significant upregulation of *Neurl3*, *Stap1*, *Cirbp,* and *Hist1h1c* and downregulation of *Tfec*, *Mitf*, *Gmds,* and *Htr2c* in the myeloid cluster of mice exposed to differently flavored e-cig aerosols as compared to air control **(Supplementary Figure 6A)**. On analyzing the lymphoid cluster for commonly dysregulated genes, we identified - *Klra8* (Killer cell lectin-like receptor 8) and *Nfia* (nuclear factor I)- that were significantly upregulated in the exposure groups as compared to air-controls **(Figure S6A)**. Klra8 is a natural killer cell associated gene, and its upregulation is generally associated with viral infection associated host immune responses within the mouse lungs (36–38). *Nfia* is a transcriptional activator responsible for regulating Oxphos-mediated mitochondrial responses and proinflammatory pathways (39, 40).

Overall, we identified a total of 29 commonly dysregulated gene targets that were identified from five major cell clusters and performed gene enrichment analyses on the identified targets to identify the top hits (Tables 2–3). Terms like ‘negative regulation of immune system’ (*Hmgb3*/*Gpam*/*Scgb1a1*/*Stap1*/*Ldlr*), ‘positive regulation of lipid biosynthetic pathway’ (*Htr2c*/*Gpam*/*Ldlr*), and ‘receptor recycling’ (*Ldlr*/*Ramp3*) were amongst the top hits in our observations **(Figure S6B)**.

Of note, the data presented in this study is a sub-part of a larger study. In addition to the groups mentioned in this manuscript, we also had two additional groups of Tobacco-Derived (TDN) and Tobacco-Free Nicotine (TFN). Though further objectives and experimentations performed in both these studies were varied, common air and PG:VG samples were used for analyses of cytokine/chemokine and cell count data as described in our recent publication(41).

## Discussion

E-cigs and associated products have constantly been under scrutiny by the US Food and Drug Administration (FDA) due to public health concerns. In February 2020, FDA placed a regulation on all cartridge-based flavored e-cigs except for menthol and tobacco to reduce the use of e-cigs amongst adolescents and young adults. But it left a loophole for the sale of flavored (including menthol) disposable and open system e-cigs (1, 42). Importantly, most e-cig related bans in the US happened at the state level, thus allowing differential levels of restrictions imposed on the premarket tobacco applications (PMTAs) and sales which defeats the purpose of limiting their accessibility to the general public (43, 44). In fact, the use of nicotine containing e-cigs amongst youth and associated policy restrictions have recently been found to be linked to unintended increase in traditional cigarette use (45). Each year new products are introduced in the market with newer device designs and properties, to lure the users (adults between the ages of 18–24 years) which makes it crucial to continue with the assessments of toxicity and health effects of e-cigs in an unbiased manner (46).

Numerous studies indicate increased oxidative stress, DNA damage, and loss of neutrophil function due to exposure to e-cig aerosols *in vitro* and *in vivo* (7, 41, 47–50). However, we do not have much knowledge about the cell populations and biological signaling mechanisms that are most affected upon exposure to differently flavored e-cig aerosols at a single-cell level. To bridge this gap in knowledge, we studied the transcriptional changes in the inflammatory responses due to acute (1-hr nose-only exposure including 120 puffs for 5-consecutive days) exposure to fruit-, menthol- and tobacco-flavored e-cig aerosols using single-cell technology.

Reports indicate that the release of metal ions due to the burning of metal coil is a major source of variation during e-cig exposures (51–53). A 2021 study reported the presence of 21 elements in the pod atomizers from different manufacturers identifying a high abundance of 11 elements including nickel, iron, zinc, and sodium amongst others (54). Previous studies have also shown the presence of similar elements in the e-cig aerosols which could have a possible adverse health effect on the vapers (19, 55). More importantly, our study points towards a much important issue, which pertains to the product design of the e-cig vapes. We believe that the aerosol composition varies based on the type of atomizer, coil resistance, coil composition, and chemical reactivity of the e-liquid being used. While much work is done on the chemical composition of the flavors and e-liquids, the other aspects of device design remain understudied and must be an area of research in the future. Another factor that may limit the interpretation of our results pertains to the correlation between the leached metal and the observed transcriptional changes. Since our study provides proof of day-to-day variation in the leaching of metal ions from the same liquid using the same atomizer, it could be possible to develop a statistical model correlating the differential metal exposure to the gene expression changes. We do not conduct such analyses as this was not the focus of this work, but it is a possibility that could be explored in the future.

E-cig vaping has been known to affect the innate and adaptive immune responses among vapers (49, 56–58), but flavor-specific effects on immune function are not fully explored. While we expected to see flavor-dependent changes in our experiment, we did not anticipate observing interesting sex-dependent variation in the lung tissues at single-cell level. In this respect, recent studies show concurring evidence suggesting sex-specific changes in lung inflammation, mitochondrial damage, gene expression, and even DNA methylation in mice exposed to e-cig aerosols (59, 60).

One of the most interesting discoveries from our single-cell analyses was the identification of a cluster of immature neutrophils without Ly6G surface marker. While we observed an increase in the cell percentages of these cells in our treatment groups, little to no change in the gene expression was noted which could be indicative of impaired function of these neutrophil population, a fate being reported by various previous studies pertaining to e-cig exposures (13, 56). In fact, our study identified an increase in the level of both mature (Ly6G+) and immature (Ly6G−) neutrophils in tobacco-exposed mouse lung which was corroborated by (a) increase in staining for Ly6G − and Ly6G+ neutrophils in female, but not male mice, through immunofluorescence, and (b) mild to no change in the levels of IL-1β in the lung tissue digests from exposed mice as compared to air. Ly6G is an important marker of neutrophil maturation in mammalian cells and has been reported in relation to various bacterial and parasitic infections in previous studies (61, 62).Exposure to tobacco flavored e-cig aerosols may result in a loss of function of mature neutrophils in the mouse lungs which gets replaced by inactive/immature neutrophils that are deficient in Ly6G surface marker for maturation. In fact, this could explain our inability to identify significant changes in mature neutrophil population through flow cytometry (where Ly6G was used as a marker) when comparing treated and control groups. Interestingly, our *in vivo* findings were corroborated by a recently published work where neutrophils from healthy volunteers demonstrated a reduction in neutrophil chemotaxis, phagocytic function, and neutrophil extracellular trap formation on exposure to e-cig aerosols, thus suggesting loss of function by mature neutrophils (49). However, this study did not investigate the effects on the immature neutrophil population, an area that needs further study in future research.

Of note, myeloid and lymphoid limbs of immunity are interconnected (63, 64). A decline in the neutrophil-mediated immunity might activate other cell types to offer protection. In our study, we find a decline in the neutrophilic immune responses in menthol and tobacco flavored e-cig exposed mouse lungs. Contrarily, we report an increase in the T-cell responses in the form of increased CD8+ T cells from both scRNA seq and flow cytometric analyses in male mice. In fact, increased expression of genes including *Malt1*, *Serpinb9b,* and *Sema4c* are indicative of enhanced T-cell mediated immune response in the lungs of mice exposed to fruit(mango) flavored e-cig aerosols (65–67). Contrary to this, exposure to menthol-flavored e-cig aerosols had a much milder effect on the lymphoid population within the lungs of C57BL/6J mice. We found evidence for increased lymphoid cell proliferation due to activation of cyclin-dependent protein kinase signaling mediated via expression of genes including *Cdk8* and *Camk1d* in these cells (33, 68). Exposure to tobacco-flavored e-cig aerosol provided evidence for decreased chaperone-mediated protein folding, due to the downregulation of Chaperonin Containing TCP-1 (CCT) family of proteins. Chaperonin Containing TCP-1 proteins are important to regulate the production of native active, tubulin, and other proteins crucial for cell cycle progression and cytoskeletal organization (69). This is in conjunction with the upregulation of *Klra4* and *Klra8* that is indicative of increased protein misfolding and cytotoxic responses in the lymphoid cells of tobacco-exposed e-cig aerosols (70, 71).

Overall, we provide evidence of subdued innate immune responses due to loss of function of neutrophils and increased T-cell proliferation and cytotoxicity in a flavor-dependent manner upon exposure to e-cig aerosols in this study. An increase in the levels of CCL17, CCL20, CCL22, IL2 and Eotaxin in the lung digests from tobacco-exposed mouse lungs further support this deduction as these cytokines/chemokines are associated with T-cell mediated immune responses (72–79). Importantly CXCL16 attracts T-cells and natural killer cells to activate cell death. It is involved in LPS-mediated acute lung injury (ALI), an outcome which has also been linked with e-cig exposures in human (80, 81).

Further, we compiled a list of commonly dysregulated genes in a flavor independent manner and identified 29 gene targets. Signal-transducing adaptor protein-2 (*Stap1)* which is commonly upregulated upon exposure to e-cig aerosols is known to regulate T-cell activation and airway inflammation which is in agreement with the overall outcome of our findings (82). Another gene that was found to be consistently dysregulated in many cell types was Cold-inducible RNA binding protein (*Cirbp*). *Cirbp* is a stress response protein linked with stressors like hypoxia. Its upregulation upon e-cig exposure supports that vaping induces oxidative stress and can have adverse implications on the exposed cell types (83). 5-hydroxytryptamine receptor 2C (*Htr2c*) and *Klra8* are other genes in this category of commonly dysregulated genes that are associated with enhancing inflammation and cell death (70, 71, 84). Overall, we provide a cell-specific atlas of immune responses upon exposure to differently flavored e-cig aerosols.

Considering that scRNA technology has not been commonly used for e-cig research, ours is one of the first studies employing this technique to identify possible changes in the cellular composition and gene expressions. Importantly we use the nose-only exposure system for our experiment to avoid exposure through other routes. However, despite the novel approach and state-of-the-art exposure system, we had a few limitations. First, we used a small sample size to identify the changes in the mouse lungs upon exposure to e-cig aerosols at a single-cell level. Due to the expensive nature of single-cell sequencing technology and limited information in the literature reporting changes at single-cell level, we chose to design this experiment with small sizes of experimental and validation cohort. But, based on the encouraging findings from this study, future studies could be designed with a larger sample size,longer durations of exposure and more targeted approach to identify the acute and chronic effects of vaping *in vivo*. Second, we could not expand upon the sex-dependent changes observed through our work upon exposure. This was because such an effect was not anticipated when we conceived the idea of a short-term exposure in mice. However, considering the evidence from the cuttenr study, future experimental designs in our lab will be designed taking sex as a crucial confounder for studying the effects of e-cig exposure in translational contexts. Third, the inclusion of PG:VG + Nic group was streamlined in this study, but in future work including this group for scRNA seq analyses might be crucial to delineate the effects of nicotine alone on gene transcription. Fourth, we did not anticipate changes in the metal release on consecutive days of exposure at the start of our study. Later, our data pointed towards the importance of device design in e-cig exposures. Future studies need to identify the factors that may affect the daily composition of e-cig aerosols and devise a method of better monitoring these possible confounders. However, in this regard our experiment does mimic the real-life scenario, as such variations due to prolonged storage of e-liquid and differences arising due to vape design must be common amongst human vapers.

In conclusion, we identified cell-specific changes in the gene expressions upon exposure to e-cig aerosols using single cell technology. We identified a set of top 29 dysregulated genes that could be studied as markers of toxicity/immune dysfunction in e-cig research. Future work with larger sample sizes and sex-distribution is warranted to understand the health impacts of long-term use of these novel products in humans.

## Methods

### Animals Ethics Statement

All experiments were conducted per the guidelines set by the University Committee on Animal Resources at the University of Rochester Medical Center (URMC). Care was taken to implement an unbiased and robust approach during the experimental design and conduction of each experiment to ensure data reproducibility per the National Institutes of Health standards.

### Animals

We ordered 5-week-old pups of male and female C57BL/6J mice from Jackson Laboratory to conduct this experiment. Prior to the start of the experiment, mice were housed at the URMC Vivarium for acclimatization. Thereafter the animals were moved to the mouse Inhalation Facility at URMC for training and exposures.

One week prior to the start of the exposures, mice underwent a five-day nose-only training to adapt themselves to the mesh restraints of the exposure tower. The mouse restraint durations were increased gradually to minimize the animal’s stress and discomfort. Of note, the mouse sacrifice was performed within 8–12 weeks’ age for each mouse group to ensure that the mouse age corresponds to the age of adolescents (12–17 years) in humans (85, 86). Age and sex-matched animals (n = 2/sex/group) used to perform single cell RNA sequencing (scRNA seq) were considered as the ‘experimental cohort’; whereas another group of age and sex-matched (n = 3/sex/group) mice exposed to air and flavored e-cig aerosol served as ‘validation cohort’ for this study.

### E-cigarette Device and E-liquid

We utilized an eVic-VTC mini and CUBIS pro atomizer (SCIREQ, Montreal, Canada) with a BF SS316 1.0-ohm coil from Joyetech (Joyetech, Shenzhen, China) for vaping and the inExpose nose-only inhalation system from SCIREQ (SCIREQ, Montreal, Canada) for mouse exposures. Both air and PG:VG exposed mice groups were considered as controls for this experiment. We used commercially available propylene glycol (PG; EC Blend) and vegetable glycerin (VG; EC Blend) in equal volumes to prepare a 50:50 solution of PG:VG. For flavored product exposures, mice were exposed to three different e-liquids – a menthol flavor “Menthol-Mint”, a fruit flavor “Mango” and a tobacco flavor “Cuban Blend”. Of note, all the e-liquids were commercially manufactured with 50mg/mL of tobacco derived nicotine (TDN). So, all treatments have nicotine in addition to the flavoring mentioned respectively. Additionally, we used a mixture of PG:VG with 50mg/mL of TDN as a control for limited experiments to study the effect of nicotine alone in our treatment. This group is labeled as PG:VG+Nic for the rest of the manuscript.

### E-cigarette Exposure

Scireq Flexiware software with the InExpose Inhalation system was used for controlling the Joyetech eVic-VTC mini device to perform nose-only mouse exposures. For this exposure, we utilized a puffing profile that mimicked the puffing topography of e-cig users in two puffs per minute with a puff volume of 51 mL, puff duration of 3 seconds, and an inter puff interval of 27 seconds with a 2 L/min bias flow between puffs (87). Figures 1A & **S1A** depict the experimental design and exposure system employed for this study.

Age-matched male and female (n=5 per sex) mice were used for each group, namely, air, PG:VG, Fruit, Menthol, and Tobacco. To ensure rigor and reproducibility in our work, we have used age- and sex-matched control and treated mice in this study. Confounders like environment and stress were minimized by housing all the cages in environmentally controlled conditions and training all the mice (both control and treated) in nose-only chambers. Each group of mice was exposed to the above-mentioned puffing profile for one hour each day (120 puffs) for a total of five consecutive days. Additionally, a group of mice (n=3/sex) was exposed to PG:VG + Nic for the same duration using similar exposure profile to serve as control to assess the effect of nicotine on the observed changes using selected experiments. Air-exposed mice were exposed to the same puffing profile for a total of five consecutive days to ambient air. We recorded the temperature, humidity, and CO levels of the aerosols generated at the start, mid, and end of the exposure on each day using the Q-Trak Indoor Air Quality Monitor 7575 (TSI, Shoreview, MN). Total Particulate Matter (TPM) sampling was done from the exhaust tubing of the set-up at the 30 min mark of the exposure and at the inlet connected to the nose-only tower (shown in **Figure S1A**) immediately after the culmination of the exposure. Gravimetric measurements for TPM were also conducted to confirm relative dosage to each mouse group daily.

### Preparation of single-cell suspension

The animals were sacrificed (at 8–10 weeks’ age) immediately after the final exposure. Vascular lung perfusion was performed using 3 mL of saline before harvesting the lung lobes for preparation of single-cell suspension. It is important to mention here that of the 5 lung samples/sex/group; 2 sex/group were used for histological assessments and scRNA analyses. Here, the left lung lobe was inflated using low-melting agarose and used for histology, while the rest of the uninflated lung lobes were used for preparing the single-cell suspension. We pooled the lung lobes for each sex per group for preparation of the single-cell suspension as depicted in Figure 1A. The lung lobes were weighed digested using Liberase method as described earlier (88). Briefly, lung lobes were weighed and digested using Liberase (Cat# 5401127001; Roche, Basel, Switzerland) enzymatic cocktail with 1% DNase. The tubes were then transferred to the gentleMACS dissociator (Miltenyi, Gaithersburg, MD) and the manufacturer’s protocol for mouse lung digestion was run. The sample tubes were next incubated at 37°C for 30 minutes with constant rotation after which the suspension was strained through 70-micron MACS Smart Strainer. Thereafter, the suspension was centrifuged at 500g for 10 minutes at 4°C, the supernatant was discarded, and 0.5 mL of RBC lysis buffer was added to the cell pellet to digest RBCs. The suspension was left on ice for 5 min in RBC lysis buffer and then 4 mL of ice-cold PBS with 10% FBS was added to stop the lysis. The suspension was again centrifuged at 500g for 10 minutes at 4°C. The cell pellet was suspended in 1 mL PBS with 10% FBS, and cell number and viability were checked using AO/PI staining on a Nexcelom Cellometer Auto2000.

### Library Preparation and Single-Cell Sequencing

The prepared single-cell suspension was sent to the Genomics Research Center (GRC) at URMC for library preparation and single-cell sequencing. Library preparation was performed from control and treatment groups using the 10X single cell sequencing pipeline by 10X Genomics and 10,000 cells were captured per sample using the Chromium platform. The prepared library was sequenced on NovaSeq 6000 (Illumina, San Diego, CA) at a mean sequence depth of 30,000 reads per cell. Read alignment was performed to GRCm38 Sequence.

### Data Analyses

We used the standard Seurat v4.2 analyses pipeline to analyze our data (89). In brief the low-quality cells and potential doublets were excluded from the dataset to create the analyses dataset. The residual features due to the presence of RBCs were corrected before integration. “scTransform” function was used for integration of all the datasets after which the standard Seurat pipeline was used for data normalization of integrated data. “FindVariableGenes” gene function was used to identify the variable genes for dimensionality reduction using PCA function. UMAP was used for dimensionality reduction and clustering of cells.

To identify the unique features and cell clusters within each cell subtype, we used the sub-setting feature within Seurat. After identifying the 5 major cell populations (epithelial, endothelial, stromal, myeloid and lymphoid) in our data sets, each of these cell types were sub-clustered using “subset” function, normalized and re-clustered. Cell annotation for each of the subsets was performed with the help of Tabula Muris database (90, 91). However, some clusters were annotated manually with the help of a literature search as discussed in the results section.

DESeq2 (V. 1.42.1) was used to perform pseudobulk analyses to identify differentially expressed genes within each group. The ClusterProfiler R package (V. 4.10.1) (92) was employed to perform gene enrichment analyses of the differentially expressed genes.

### Cytokine/chemokine assessment

We used multiplex assay to determine the levels of cytokine/chemokine in the lung homogenates from control and e-cig aerosol exposed mouse lungs using commercially available Bio-Plex Pro Mouse Chemokine Assay (Cat#12009159, Bio-RAD, Hercules, CA) per the manufacturer’s instructions. Approximately 40 mg of mouse lung lobes were homogenized in 300uL of 1X RIPA buffer with 0.1% protease and phosphatase inhibitor. The lung homogenate was stored on ice for 30 min. Following incubation, the homogenate was centrifuged at 15000 rpm for 15 min at 4 degrees Celsius. The supernatant was collected and used for performing the multianalyte assay for determination of cytokine/chemokine levels using Luminex FlexMap3D system. A heatmap after normalization of the measured cytokine/chemokine to the protein amount loaded was plotted.

### Lung Histology

The left lung lobe of mice used for scRNA seq were inflated with 1% low melting agarose and fixed with 4% neutral buffered PFA. Fixed lungs were dehydrated, before being paraffin‐embedded and sectioned (5μm). Hematoxylin and eosin (H&E) staining was performed by the Histology, Biochemistry, and Molecular Imaging Core at URMC. The H&E stain was observed at 10X magnification using Nikon Elipse‐Ni fluorescence microscope. Ten to fifteen random images were captured per sample.

### Flow cytometry

Flow cytometry was performed on the cells collected from lung homogenates from air and flavored e-cig aerosol exposed mouse lungs. For analyses of immune cell population in the lung, the lung lobes were digested as described earlier (88) . The single-cell suspension thus prepared was used to run flow cytometry using the BD LSRFortessa cell analyzer. Cells were blocked with CD16/32 (Tonbo biosciences 70–0161-u500, 1:10) to prevent nonspecific binding and stained with a master mix of Siglec F (BD OptiBuild Cat#740280, 1:200), CD11b (Biolegend Cat #101243, 1:200), Ly6G (BD Horizon Cat# 562700, 1:200), CD45 (Biolegend Cat#103126, 1:200), CD11c (Biolegend Cat #117318, 1:200), CD4 (Biolegend Cat#116012, 1:200), and CD8 (eBiosciences Cat#17–0081-82, 1:200). 7AAD (eBiosciences Cat#00–6993-50, 1:10) was used as the nucleic acid dye to detect live and dead cells.

### Metal analyses

To understand the levels of metals released during subsequent days of exposure, we performed Inductively coupled plasma mass spectrometry (ICP-MS) on the e-cig aerosol condensates collected from each day of exposure using a Perkin Elmer ICP-MS model 2000C. The samples were run using a Total Quant KED protocol with 4mL/min Helium flow and externally calibrated using a blank and a 100ppb standard for the 51 elements. The samples were submitted to the Element Analyses facility at URMC, and levels of metals thus detected were plotted.

### Ly6G /S100A8 Double staining

To determine the presence of Ly6G+ and Ly6G− neutrophils, FFPE tissue sections from air and tobacco-flavored e-cig aerosol exposed lungs were stained with Ly6G and S100A8. In brief, 2–3 tissue sections per sample were deparaffinized using serial incubation in xylene followed by graded alcohol. Slides were incubated in 1X Citrate Buffer (Cat# S1699, Agilent, Santa Clara, CA) for 10 min at 95°C for antigen retrieval which was followed by incubation at room temperature for 30 min. The slides were next washed with water and permeabilized using a permeabilization buffer (0.1% Triton-X in 1X TBST) for 10 min. Next, the slides were again washed with 1X TBST and blocked using Blocking buffer (5% goat serum in 1X TBST) for 30 min at room temperature. The blocked slides were incubated overnight at 4°C with Ly6G (Cat# 16–9668-85, Invitrogen, dilution: 1:100) and S100A8 (Cat# 26992–1-AP, Proteintech, dilution: 1:200). Next day, the slides were washed with 1X TBST and incubated for 2 hrs. at room temperature with goat anti-rabbit Alexa Fluor 594 (Cat # A11012, Invitrogen) and donkey anti-mouse Alexa Fluor 488 (Cat # A21202, Invitrogen) secondary antibody at 1:1000 dilution. Thereafter the slides were washed and mounted with ProLong Diamond Antifade Mountant with DAPI (Cat# P36962, Invitrogen, Waltham, MA). 6–10 images were captured using Cytation 5 Imaging software (Thermo Fisher, Waltham, MA) at 10X magnification.

### Statistical significance

We used GraphPad Prism 10.5.0 for all statistical calculations. All the data plotted in this paper are expressed as mean ± SEM. Pairwise comparisons were done using unpaired *t* test while one‐way analysis of variance (ANOVA) with ad‐hoc Tukey’s test was employed for multi-group comparisons. To identify sex-based variations in our treatment groups, Tukey post hoc two-way ANOVA was employed.

## Figures and Tables

**Table 1:** Table showing the levels of common elements found in the flavored e-liquids and e-cig aerosols as measured using ICP-MS.

Eleme nt	Eliquid; ppb/ mg of eliquid				E-cig Aerosol (Mean ± SD); ppb/ mg of eliquid			
	PG:VG	Fruit	Menthol	Tobacco	PG:VG	Fruit	Menthol	Tobacco
S	75.63	68.36	95.34	79.79	70.67 ± 16.94	74.05 ± 39.63	81.31 ± 27.24	91.81 ± 11.67
Ni	2.09	1.63	4.73	2.84	1.04 ± 1.00	30.47 ± 14.28	83.52 ± 20.53	41.73 ± 12.25
Cu	2.16	0.87	2.72	2.00	0.37 ± 0.22	1.04 ± 0.48	1.85 ± 0.70	1.32 ± 0.39
Si	1.05	1.13	1.55	1.26	1.10 ± 0.14	1.09 ± 0.35	1.32 ± 0.28	1.33 ± 0.12
K	0.80	0.84	1.21	0.67	0.94 ± 0.13	0.99 ± 0.45	1.32 ± 0.28	0.51 ± 0.10
Na	0.39	0.40	1.10	0.63	0.65 ± 0.11	0.91 ± 0.17	1.70 ± 0.37	0.79 ± 0.11
W	0.48	0.23	0.24	0.14	0.46 ± 0.20	0.30 ± 0.18	0.28 ± 0.14	0.22 ± 0.05
Zn	0.32	0.36	0.68	1.94	0.18 ± 0.01	1.45 ± 0.48	2.06 ± 0.48	1.71 ± 0.42
Ir	0.28	0.47	0.27	0.11	1.19 ± 0.76	0.35 ± 0.29	0.19 ± 0.12	0.14 ± 0.05
B	0.14	0.02	0.02	0.02	0.07 ± 0.02	0.02 ± 0.01	0.02 ± 0.01	0.02 ± 0.01
Ta	0.14	0.34	0.24	0.13	0.73 ± 0.30	0.24 ± 0.09	0.18 ± 0.06	0.13 ± 0.02
Hf	0.13	0.18	0.13	0.07	0.43 ± 0.19	0.14 ± 0.05	0.09 ± 0.03	0.07 ± 0.01
Mo	0.08	0.01	0.01	0.01	0.02 ± 0.01	0.07 ± 0.01	0.15 ± 0.04	0.06 ± 0.02
Pd	0.07	0.14	0.11	0.06	0.30 ± 0.14	0.11 ± 0.06	0.07 ± 0.02	0.06 ± 0.01
Pt	0.05	0.06	0.05	0.07	0.16 ± 0.07	0.09 ± 0.05	0.08 ± 0.04	0.08 ± 0.03
Zr	0.04	0.01	0.02	0.02	0.02 ± 0.01	0.02±0.01	0.01 ± 0.00	0.01 ± 0.00
Sn	0.02	0.06	0.05	0.04	0.06 ± 0.02	0.07 ± 0.02	0.03 ± 0.01	0.03 ± 0.01
Mg	0.01	0.01	0.04	0.03	0.02 ± 0.00	0.12 ± 0.03	0.12 ± 0.03	0.10 ± 0.02
Al	0.01	0.01	0.01	0.01	0.01 ± 0.00	0.01 ± 0.00	0.01 ± 0.00	0.01 ± 0.00
Co	0.01	0.01	0.01	0.01	0.01 ± 0.00	0.02 ± 0.00	0.03 ± 0.01	0.02 ± 0.00
Nb	0.01	0.00	0.00	0.00	0.01 ± 0.00	0.00 ± 0.00	0.00 ± 0.00	0.00 ± 0.00
Ba	0.01	0.01	0.01	0.01	0.01 ± 0.01	0.01 ± 0.00	0.01 ± 0.00	0.01 ± 0.00
Re	0.01	0.01	0.01	0.02	0.06 ± 0.04	0.03 ± 0.03	0.02 ± 0.01	0.02 ± 0.01

**Table 2:** List of top dysregulated genes on exposure to differently flavored (fruit, menthol and tobacco) e-cig aerosol in C57BL/6J mouse lungs

Genes	Gene Names	Gene Function
Neurl3	Neuralized E3 Ubiquitin Protein Ligase 3	ubiquitin protein ligase activity
Egfem1	EGF-like and EMI domain containing 1	calcium ion binding activity
Stap1	Signal Transducing Adaptor Family Member 1	protein kinase binding and SH3/SH2 adaptor activity
Tfec	Transcription Factor EC	multiple cellular processes including survival, growth and differentiation
Mitf	Melanocyte Inducing Transcription Factor	critical role in cell differentiation
Cirbp	Cold Inducible RNA Binding Protein	role in cold-induced suppression of cell proliferation
Hist1h1c	H1.2 linker histone	functions in the compaction of chromatin
Gmds	GDP-Mannose 4,6-Dehydratase	coenzyme binding and NADP^+^ binding
Htr2c	5-Hydroxytryptamine Receptor 2C	G protein-coupled receptor activity
Nfia	Nuclear Factor I A	DNA-binding transcription factor activity
Klra8	killer cell lectin-like receptor	carbohydrate binding activity. Acts upstream of or within response to virus
Trp53i11	Tumor Protein P53 Inducible Protein 11	negative regulation of cell population proliferation
Ehd2	EH Domain Containing 2	Angiopoietin-like protein 8 regulatory pathway and response to elevated platelet cytosolic Ca2+.
Ackr2	Atypical Chemokine Receptor 2	recruitment of effector immune cells to the inflammation site.
Marcks	Myristoylated Alanine Rich Protein Kinase C Substrate	involved in cell motility, phagocytosis, membrane trafficking and mitogenesis
Pfkl	Phosphofructokinase,	protein binding and monosaccharide binding
Ramp3	Receptor Activity Modifying Protein 3	signaling receptor activity and coreceptor activity
Chrm3	Cholinergic Receptor Muscarinic 3	cellular responses such as adenylate cyclase inhibition, phosphoinositide degeneration, and potassium channel mediation
Sftpal	Surfactant Protein A1	carbohydrate binding and lipid transporter activity
Add3	Adducin 3	actin binding and calmodulin binding
Hmgb3	High Mobility Group Box 3	important role in maintaining stem cell populations and may be aberrantly expressed in tumor cells.
Acot1	Acyl-CoA Thioesterase 1	involved in acyl-CoA metabolic process; long-chain fatty acid metabolic process; and very long-chain fatty acid metabolic process
H1f0	H1.0 Linker Histone	Cellular responses to stimuli and Programmed Cell Death.
Scgb3a2	Secretoglobin Family 3A Member 2	secreted lung surfactant protein
Scgb1a1	Secretoglobin Family 1A Member 1	implicated in numerous functions including anti-inflammation, inhibition of phospholipase A2 and the sequestering of hydrophobic ligands
Gpam	Glycerol-3-Phosphate Acyltransferase, Mitochondrial	acyltransferase activity and glycerol-3-phosphate O-acyltransferase activity
Cdh11	Cadherin 11	integral membrane proteins that mediate calcium-dependent cell-cell adhesion.
Ldlr	Low Density Lipoprotein Receptor	cell surface proteins involved in receptor-mediated endocytosis of specific ligands.
Myocd	Myocardin	transcriptional co-activator of serum response factor (SRF)

**Table 3:** Gene Ontology results showing the top hits from the commonly dysregulated genes in all clusters on exposure to e-cig aerosols

GO ID	ONTOLOGY	Description	Gene ID	BgRatio	p.adjust
GO:0045907	BP	positive regulation of vasoconstriction	Htr2c/Chrm3/Add3	53/23062	0.033815
GO:0019229	BP	regulation of vasoconstriction	Htr2c/Chrm3/Add3	86/23062	0.042491
GO:1903978	BP	regulation of microglial cell activation	Stap1/Ldlr	16/23062	0.042491
GO:0002683	BP	negative regulation of immune system process	Hmgb3/Gpam/Scgb1a1/Stap1/Ldlr	464/23062	0.042491
GO:0010867	BP	positive regulation of triglyceride biosynthetic process	Gpam/Ldlr	19/23062	0.042491
GO:0042310	BP	vasoconstriction	Htr2c/Chrm3/Add3	109/23062	0.042491
GO:0046889	BP	positive regulation of lipid biosynthetic process	Htr2c/Gpam/Ldlr	110/23062	0.042491
GO:0010866	BP	regulation of triglyceride biosynthetic process	Gpam/Ldlr	25/23062	0.047046
GO:0001919	BP	regulation of receptor recycling	Ldlr/Ramp3	29/23062	0.047046
GO:000771	BP	synaptic transmission, cholinergic	Htr2c/Chrm3	29/23062	0.047046
GO:0150077	BP	regulation of neuroinflammatory response	Stap1/Ldlr	29/23062	0.047046
GO:0090208	BP	positive regulation of triglyceride metabolic process	Gpam/Ldlr	31/23062	0.047046
GO:0045987	BP	positive regulation of smooth muscle contraction	Chrm3/Myocd	35/23062	0.047046
GO:0097242	BP	amyloid-beta clearance	Ldlr/Myocd	36/23062	0.047046
GO:0040013	BP	negative regulation of locomotion	Htr2c/Mitf/Stap1/Myocd	360/23062	0.047046
GO:0010667	BP	negative regulation of cardiac muscle cell apoptotic process	Acot1/Myocd	37/23062	0.047046
GO:0001881	BP	receptor recycling	Ldlr/Ramp3	40/23062	0.047046
GO:0010664	BP	negative regulation of striated muscle cell apoptotic process	Acot1/Myocd	40/23062	0.047046
GO:0019432	BP	triglyceride biosynthetic process	Gpam/Ldlr	40/23062	0.047046
GO:0035296	BP	regulation of tube diameter	Htr2c/Chrm3/Add3	173/23062	0.047046
GO:0097746	BP	blood vessel diameter maintenance	Htr2c/Chrm3/Add3	173/23062	0.047046
GO:0001774	BP	microglial cell activation	Stap1/Ldlr	42/23062	0.047046
GO:1903725	BP	regulation of phospholipid metabolic process	Htr2c/Ldlr	42/23062	0.047046
GO:0035150	BP	regulation of tube size	Htr2c/Chrm3/Add3	174/23062	0.047046

## Data Availability

The datasets generated during and/or analyzed during the current study are deposited on NCBI Gene Expression Omnibus. Specifically, mouse scRNAseq is available under accession code “GSE263903” (https://www.ncbi.nlm.nih.gov/geo/query/acc.cgi?acc=GSE263903). The data will be publicly available upon publication or on April 11, 2026, whichever is earlier. Reviewer’s token for access is ‘wtofqmayjdeltcn’. All other relevant data supporting the key findings of this study are available within the article and its Supplementary Information files or from the corresponding author upon reasonable request. Source data are provided with this paper.
